# Implementation of a Family Centered Telecoaching Intervention for Parents of Children with Motor Difficulties: A Multimethod Process Evaluation

**DOI:** 10.63144/ijt.2025.6722

**Published:** 2025-12-12

**Authors:** Karen Hurtubise, Isabelle Gaboury, Chantal Camden, Rosalie Dostie, Audrée Jeanne Beaudoin, Désirée Maltais, Meaghan Reitzel, Jade Berbari, Mélanie M. Couture, Mélanie M. Morin, Michelle Phoenix

**Affiliations:** 1 Faculté de Médecine et Sciences de la Santé, Université de Sherbrooke, Sherbrooke, Québec, Canada; 2 Centre de recherche du Centre Hospitalier Universitaire de Sherbrooke, Sherbrooke, Québec, Canada; 3 CanChild Centre for Childhood Disability Research, McMaster University, Hamilton, Ontario, Canada; 4 School of Rehabilitation Sciences, Faculty of Health Sciences, McMaster University, Hamilton, Ontario, Canada; 5 Centre Intégré Universitaire de Santé et de Sciences Sociaux de l’Estrie-Centre Hospitalier Universitaire de Sherbrooke, Sherbrooke, Québec, Canada; 6 École de sciences de réadaptation, Faculté de Médecine, Université Laval, Québec, Québec, Canada

**Keywords:** Coaching, Intervention fidelity, Multimethod design, Pediatric rehabilitation, Process evaluation

## Abstract

This multimethod process evaluation aimed to explore the implementation of a telehealth coaching intervention for parents of children with motor difficulties. Four therapists and 59 parents participated. The dosage of 525 sessions was compared to the study protocol. Thirty-three external rater assessments, 62 therapists’ self-adherence and 59 parent satisfaction surveys were analyzed descriptively for adherence, parent responsiveness and intervention quality. Therapists’ interviews were analyzed thematically for implementation experience. A median of nine sessions per family was provided; only 58% were received within the prescribed timeframe. Adherence (83%), participant responsiveness (91%), and intervention quality (85%) were high, along with therapists’ self-adherence (84%) and parent satisfaction (87%). Therapists reported partnering effectively with parents in the intervention and maintaining a family-oriented approach. Regular feedback and mentorship were the most effective implementation strategies identified by therapists. Telehealth coaching interventions can be implemented with high fidelity when therapists receive proper training and support.

One to two children in classrooms present with a motor difficulties (Klein et al., 2024), some with a diagnosis (e.g., Development Coordination Disorder), others without. These difficulties create functional challenges for the child across developmental spheres (e.g., social, cognitive, behavioural) (Gosse et al., 2024). In addition to the impact on the child, these challenges also can negatively affect parents (e.g., increased parental stress, depression and anxiety) and families (e.g., ruptured family unit) (Dhiman et al., 2020; Feldman et al., 2008).

Early access to occupational and physiotherapy services can help children with motor difficulties achieve motor skills (Gmmash et al., 2021). However, currently, worldwide healthcare service models, including those used in pediatric rehabilitation services, are not nimble enough and do not have a broad enough reach to address these challenges, resulting in long wait times and service gaps (Gosse et al., 2024; Klein et al., 2024; Ogourtsova, 2019). These disparities are of particular concern for families living rurally and remotely, where children are four times more likely to experience development concerns (Gosse et al., 2024) and access to the services they need is limited (Gerlach et al., 2023; Hines et al., 2019).

Web-based technologies provide a unique opportunity to improve access for families of children with developmental difficulties to a comprehensive continuum of healthcare services, including those required to optimize child development (Camden & Silva, 2021; Hines et al., 2019; Ogourtsova et al., 2023). Contemporary family-centered practices in pediatric rehabilitation interventions acknowledge the active role of parents in goal setting, therapy implementation, and evaluation as best practice (Akhbari Ziegler, 2020; Akhbari Ziegler et al., 2019; Dunst et al., 2014; Graham et al., 2024; King et al., 2024). Furthermore, the importance and value of pediatric therapists (including physiotherapists and occupational therapists) engaging and supporting parents in implementing intervention strategies within the home and community environments have been acknowledged (Akhbari Ziegler et al., 2019; Dunst, 2020; Graham et al., 2024).

Parent coaching interventions align with these family-centred practices and involve working collaboratively with parents to improve their existing abilities, develop new skills, and enhance their problem-solving capabilities to support their child’s development and goal achievement (Akhbari Ziegler et al., 2019; Brown & Woods, 2016; Dirks & Hadders-Aalgra, 2011; Graham et al., 2024; Kemp & Turnbull, 2014; King et al., 2019). Despite the endorsement of the family-centred care philosophy across pediatric services, including those targeting rehabilitation, studies on families’ experiences with these services underscore a lack of consensus on the actions and strategies for implementing family-centered principles into practice (Angeli et al., 2019; Gao, 2023; Pozniak et al., 2024). As such, evidence suggests that a paradigm shift is required away from the health providers as the experts, where their needs and priorities dictate services, to a model where families’ concerns are listened to, their expertise is respected, and where providers work collaboratively and in partnership with them (Baldwin et al., 2013; Graham, Boland, et al., 2018; Seko et al., 2020; Ziegler et al., 2018).

Coaching interventions delivered via web-based technology (e.g., videoconferencing), also known as telecoaching, have demonstrated promise in shifting this paradigm, thereby enhancing parents’ efficacy and children’s participation in some specific pediatric populations (e.g., autism spectrum disorder; language disorders) (Camden, 2019; Graham et al., 2023; Little et al., 2018; Ogourtsova et al., 2023). However, sustained uptake of this technology for the delivery of therapeutic interventions in clinical practice has been limited, despite its high use during the COVID-19 pandemic (Graham et al., 2023). Sustained uptake of web-based interventions is influenced by a complex interplay of technology, individual, and contextual factors (Chen et al., 2024; Cottrell & Russell, 2020; Ross, 2016). Many published knowledge syntheses have underscored the broad acceptance and satisfaction of web-based interventions by families (e.g., Alonazi, 2021; Caprì et al., 2021; Dostie et al., 2022; Kodjebacheva et al., 2023; Moreno-Chaparro et al., 2025; Ogourtsova et al., 2023). However, a lack of training, skills and confidence associated with communication style and skills, safe and effective use of platforms, intervention and assessment strategies for different age groups and conditions, and a strong preference for hands-on interventions are commonly cited reasons by therapists for not sustaining the use of telehealth (Camden & Silva, 2021; Graham et al., 2023). Much of the contemporary evidence investigating telehealth adoption is based on its rapid and widespread implementation associated with the COVID-19 pandemic, which owing to the immediacy of the situation, did not follow typical implementation processes. As such, little is known about the factors impacting a planned implementation of a web-based intervention or the experience of those implementing it. This highlights a need for comprehensive evaluations to provide such insights, including their success (or failure), and how implementation processes and strategies can be improved.

Process evaluations play a key role in implementing research knowledge into standard clinical practice (Leavy et al., 2021). While the number of trials of coaching interventions in the field of childhood disabilities has grown in the past decade (Akhbari Ziegler, 2020; Baldwin et al., 2013; Graham, Ziviani, et al., 2018; King et al., 2019; Little et al., 2018; Seko et al., 2020), process evaluations in this field have been rare, in particular with children with motor difficulties. Because the provision of telecoaching interventions is still considered a novel approach in mainstream pediatric rehabilitation services, assessing their implementation in clinical settings is critical (Ward et al., 2020). Implementation information is necessary to determine whether changes in outcomes are the result of the research intervention or other factors (Carroll et al., 2007). A lack of such data renders the replicability of the intervention across therapists and settings almost impossible to assess (Di Rezze et al., 2013). Furthermore, understanding the experiences of those implementing and delivering telecoaching interventions will reveal critical lessons about implementation, refinement of the strategies used, and their generalizability across clinical contexts (Leavy et al., 2021; Moore et al., 2015).

This article presents a process evaluation of the implementation of a web-based early intervention for children using multimodAl REhabilitation (WECARE) (Camden et al., 2021), a telecoaching intervention. This study aimed to: (1) examine the implementation of the intervention, as measured using four fidelity dimensions, and (2) explore therapists’ experiences, describe any intervention modifications that occurred, and provide recommendations for future implementation.

## Materials & Methods

### Design

This multimethod descriptive process evaluation was performed as the implementation phase of a type 1 hybrid effectiveness-implementation study design (Curran et al., 2012). A previously described pragmatic randomized control trial acted as the effectiveness phase (ClinicalTrials.gov NCT04254302). Results demonstrating its effectiveness in supporting parents of children with motor difficulties will be published elsewhere. The study received ethical approval from the Research Ethics Board of the *Centre intégré de santé et des services sociaux de l’Estrie—Centre hospitalier universitaire de Sherbrooke* (identifier: 2020–3429). This process evaluation study was guided by the Medical Research Council framework for a process evaluation for complex interventions (Moore et al., 2015), the framework for evaluation of implementation fidelity (Carroll et al., 2007), and the Template for Intervention Description and Replication (Hoffmann et al., 2014).

### Intervention Description

**Figure 1:**
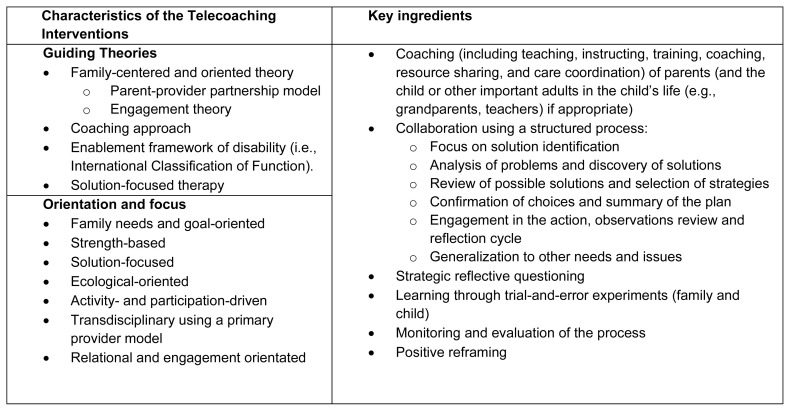

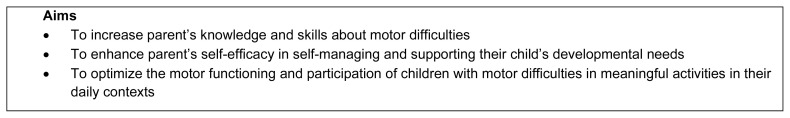
Characteristics of the Telecoaching Interventions

The Web-based Early intervention for Children using multimodAl REhabilitation (WECARE) (Camden et al., 2021), a telecoaching intervention delivered exclusively using a multi-modal web-based platform, provided 59 families of children with motor difficulties access to developmental neurorehabilitation services. The characteristics of the telecoaching intervention are presented in Figure 1, and in the previously published WECARE theory of change (Camden et al., 2021; Hurtubise et al., 2022). In summary, the intervention was designed to support parents of children with motor difficulties and focused on developing parent participants’ task analysis skills associated with their identified goals, on building their abilities to independently identify and implement strategies with their child which targeted these goals, and on reflecting on the outcome achievement.

**Figure 2:**
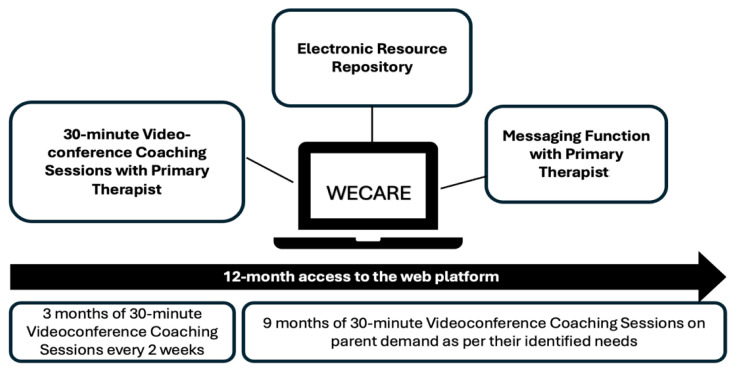
Telecoaching Intervention Delivery Features and Intervention Timeline

Before starting the telecoaching intervention, a collaborative goals identification process (Tanner et al., 2021) was completed by participating parents with a research assistant to determine study eligibility and goals to be targeted during the intervention. Participating primary therapists, either a physiotherapist or an occupational therapist, delivered the telecoaching intervention using features of the multimodal web-based platform created for the purposes of this study. Figure 2 outlines the various platform features and intervention timeline including: (1) 30-minute videoconference coaching session with the primary therapists, which targeted parent-identified goals, and was offered every two weeks to participating parents for the first 3-months of their participation in the study and at participating parents’ request for the 9-months thereafter; (2) an electronic resource repository, which therapists could use to share information and resources with families, and (3) a messaging function with the primary therapist, through which therapists and parents could communicate (e.g., ask/answer questions; problem solve; adapt strategies).

The initial individual session frequency (i.e., every two weeks for 3-months) and dosage (30-minutes per session) were selected to nurture participants’ self-confidence while supporting their problem-solving skills and helping them generalize their acquired skills (i.e., task analysis, goal refinement) and abilities (i.e., identifying and implementing strategies) to other goals and situations. Following the initial three months, session duration and frequency were adapted to parents’ needs and were scheduled upon their request until the completion of the study (i.e., total intervention duration=12 months). As flexibility is inherent to coaching approaches, no strict schedule of number or length of the sessions was instituted after three months, allowing time between sessions for parents to work with their child on the identified goals at their own pace, based on their own priorities (Camden et al., 2021).

### Context

The study occurred from March 2020 to October 2021 across the province of Quebec, Canada. Four days after the study’s rolling recruitment process began, the provincial government declared a public health emergency due to the COVID-19 pandemic. Lockdown periods occurred regularly from March 2020 to June 2021. Intermittent closures of schools, pediatric outpatient services, and non-essential workplaces were a contextual reality of this study.

### Participants

Two participant groups were involved, all of whom consented to participate.

#### Therapists

Four therapists, recruited from a pediatric rehabilitation network, delivered the intervention. To be included, therapists were required to have experience delivering child development services. Prior experience with telehealth or coaching approaches was not essential. Table 1 summarizes therapists’ sociodemographic characteristics.

None of the therapists had prior training in telecoaching. Therapists 1 and 2, recruited at the beginning of the study, provided the intervention to 90% (53/59) of the participating families. Therapists 3 and 4, recruited 4 months later to accommodate the unanticipated rapid recruitment of families, provided service to the remaining 10% (6/59). All therapists were involved until the study’s completion.

Before providing the telecoaching intervention, the four therapists received training developed by the WECARE study team. The training for participating therapists aimed to (1) introduce knowledge of coaching and develop coaching skills; (2) highlight web-based platform features; (3) teach basic technological problem-solving; and (4) review the associated study tasks and expectations (e.g., videorecording process). The training included four hours of self-paced module learning (i.e., readings and video clips) on the platform features and the key coaching behaviours, and 3.5 hours of interactive group learning activities (e.g., case example discussions, role-playing, and experience-sharing). Two therapists (i.e., Therapists 1 and 2) received their training in-person, while the other two (i.e., Therapists 3 and 4) completed the training virtually due to the pandemic restrictions. Tools to help increase the therapists’ fidelity in delivering the intervention (e.g., session scripts; checklist) were also provided. Finally, the four therapists had on demand virtual access to the principal investigator for advice. Regular mentoring meetings also occurred between the principal investigator and the four therapists, wherein performance feedback was provided, and challenges were discussed.

#### Participating Families

Using social media recruitment campaigns, family representatives (e.g., parent, legal guardian) (herein, participating parents) of children aged 3–8 years with or at risk of a motor difficulties (as determined by the Developmental Coordination Disorder Questionnaire (Parmar et al., 2014; Wilson et al., 2007)) were identified. To be eligible, representatives required functional communication abilities in English or French, and the ability to identify at least one motor goal for their child (using the Canadian Occupation Performance Measure) (Cusick et al., 2007; Law et al., 1990).

Fifty-nine parents, primarily mothers (94.9%), aged 36 to 45 years (50.8%), university-educated (74.6%), and worked fulltime (45.8%), were recruited to receive the intervention. Each lived in a nuclear family situation (84.7%), with two children (64.4%); most lived in an urban region (45.8%), with a household income of CAD 100,000 or more (39.1%). Most (59.3%) had a diagnosis which explained their child’s motor difficulties. While some children (32.2%) had previously accessed services, the majority (66.1%) were awaiting rehabilitation services. Table 2 presents a comprehensive summary of the demographic characteristics of participating families.

### Measures, Procedures, and Data Analysis

#### Intervention Quantity & Quality

Table 3 presents the dimensions used to assess the intervention implementation, their definition, the measurement selected, the procedures followed, and the data analysis strategy used. These data were collected over the 19-month intervention period and analyzed using descriptive statistics.

As outlined in Table 3, an observation checklist was created for this study for use by an external rater. A full description of the development and piloting of the *WECARE Fidelity Checklist*, as well as the rater training process and experience using the tool, are published elsewhere (Hurtubise et al., 2024). In summary, guided by the developmental process described by An et al. (2021) and Walton et al. (2020) the *WECARE Fidelity Checklist* contained three subscales: (1) the therapist behaviours subscale (12 behaviours), (2) the parent behaviours subscale (four behaviours), and (3) the parent-therapists interaction subscale (2 behaviours), ranked using a 5 point-Likert scale (see Table 4 for rating criteria).

Once a month, a trained external rater possessing pediatric clinical and research experience and expertise in parent coaching interventions, assessed two video recordings of two different therapists. The selected recordings were randomly chosen by a research team member from a pool of all the telecoaching sessions recorded during the first week of every month. As per this process, therapists were assessed on average every eight weeks, for the period during which they were providing the telecoaching sessions. Following the rater’s assessment, therapists were provided performance feedback.

#### Therapists’ Intervention Implementation Experiences

At the end of the study, in-depth semi-structured interviews were conducted with all four therapists (i.e., 100% of participants) who implemented the telecoaching intervention (See Appendix A for Interview Schedule). The purpose of these interviews was to: (1) explore participating therapists’ experiences implementing the telecoaching intervention, (2) identify if any modifications to the intervention occurred, (3) describe the modifications, the reason and circumstances under which circumstances, and (4) highlight any recommendations for future implementation of the intervention. The interviews were conducted by RD, a pediatric physiotherapist and doctoral student with formalized qualitative methodology research training, experience with conducting in-depth interviews for multiple qualitative studies, and familiarity with the telecoaching intervention. Interview recordings, which averaged 60 minutes (range 43–86 minutes), were transcribed, anonymized and analyzed thematically (Braun & Clarke, 2006). Two members of the research team, KH and MP, independently read and reread the anonymized transcripts, identifying initial codes and clustered them. KH and MP then discussed the clusters and possible themes, following which themes were named and defined. At the time of conducting the analysis, KH, a physiotherapist with clinical experience in child development, was undertaking a post-doctoral fellowship, which included involvement in the telecoaching intervention implementation. MP, a pediatric speech-language pathologist and established researcher with extensive expertise in qualitative methodology, had collaborated on the conceptualization of the telecoaching intervention but was not involved in its implementation. To establish clinical relevance and transferability, themes and their definition were reviewed with a third team member, MR, a pediatric occupational therapist, experienced in the delivery of pediatric web-based rehabilitation and qualitative analysis. A discussion followed, after which theme refinement occurred. The analysis and interpretations were reviewed by all co-authors and agreement was reached. Representative quotes are included in the themes below; numbers were assigned to protect the participant’s anonymity.

## Results

Therapists delivered 525 sessions, sent 405 messages, and spent 340 hours providing telecoaching intervention to 59 families. Thirty-three telecoaching video recorded sessions were assessed by the external rater. Participating therapists completed a self-assessment on recorded sessions (i.e., 62 sessions). The majority (84%) of recorded sessions evaluated were conducted by Therapists 1 and 2, as were self-assessments (85%) due to the higher percentage of family participants assigned to them.

### Intervention Quantity & Quality

Table 5 summarizes the assessment findings for the dimensions used to evaluate the quantity and quality of the intervention implementation.

### Dosage

A little over half (58%) of participating families received the targeted dose of the intervention (i.e., six sessions in the first three months) as outlined by the protocol. A median of nine telecoaching sessions were provided, but much variability was noted; some families availed of only one session, while others chose to continue with bimonthly sessions (as prescribed for the first three months) for their entire study participation. The median session duration was nine minutes longer than the 30 minutes proposed in the protocol. Although no parameters for use of messaging feature were prescribed, a median of five exchanges occurred with each family (range:1–20), with a median of seven minutes per message.

### Adherence

The overall score was high for the therapist’s behaviours subscale as assessed by the external rater and the therapists themselves. Therapists were rated high in their creation of a trusting relationship (81%), showing interest and attention (86%), recognition of the parent’s strengths (83%), demonstrating comfort and confidence in engaging the parent (89%), and in promoting the parent’s self-determination (89%). Behaviours graded as low included a therapists’ inquiry on the parent-identified objective to guide the session (69%), their use of positive language (61%), giving advice only when solicited (58%), summarizing the action plan (42%), and asking the parents if the objective had been met (58%) at the end of the session. Similarly, therapists also self-assessed some key behaviours higher than others, with the “session focus” item assessed as the lowest (78%), and “building relationship” (92%) as the highest.

### Participant Responsiveness

External rater’s subscale scores of parent behaviours and parent-therapist interactions were high, with some variability. Family participants’ receptiveness to the intervention (81%), their openness and willingness to participate in the session (91%), and their comfort and confidence in communicating during the session (92%) were appraised as high. On the other hand, participants’ positive orientation towards themselves and their child in the context of the intervention was rated low (62%). Parent-therapist interactive behaviours also showed some differences, with the rating for positive rapport and mutual respect being much higher (97%), than the therapist’s and parent’s mutual contribution to the session (78%). Parent’s satisfaction with the intervention rated high (87%) at the end of the study period.

### The Overall Quality of the Intervention

*A priori* quality criterion of 75% of the checklist behaviours rated as a “4” (i.e., observed regularly, but not all opportunities seized) or “5” (i.e., always observed when appropriate as intended in the intervention development), was achieved in 70% of sessions.

### Therapists’ Intervention Implementation Experiences

Three themes were generated from therapists’ interviews. The themes, their focus, a brief description and their implications for practice are highlighted in Table 6; each theme is further elucidated in the paragraphs below.

### Theme 1: Increased Collaboration with Parents

In their narratives, therapists acknowledged feeling more effective in engaging and partnering with parents in the telecoaching intervention than in the child-focused approaches previously employed in their practice. Moreover, therapists highlighted a shift from a focus on their expertise to that of parents as the experts. This therapist provided these insights:

“*What I appreciated most, was the questioning [process]. It allowed me to see the family’s skills and abilities, and telecoaching helped me better understand this*.” [Therapist 2].

Therapists also provided detailed descriptions of their interactions with parents during the study to validate their application of the essential intervention key ingredients. For example, Therapist 1 described working with a mother, who wanted to target stair mobility with her child, who tended to fall. The mother previously had the child practice going up and down a full flight of stairs. The therapist drew parallels with how the mother performed the task and used reflective questioning to support the parent in refining her therapeutic goal and understanding the principles of activity grading. Later, the mother applied the same principles to buttoning independently. This example illustrates the key ingredients of the telecoaching intervention.

### Theme 2: Remaining Family-oriented

The therapists candidly detailed instances when they deviated from the telecoaching key components, along with the circumstance and reasons why. Most often the decision to stray from the intervention components occurred when parents seemed overwhelmed by the complexities of the family’s life, the parent’s and child’s needs were high, and their expectations of health services were mismatched with the intervention. Participating therapists acknowledged that implementing some of the telecoaching components was more difficult when the parent was awaiting a diagnosis for their child and/or had a very traditional child-focused view of services (i.e., intervention provided by therapists to the child). Therapists also recognized that the intervention could be time-consuming and difficult for families to implement when parents were juggling multiple priorities (e.g., children’s schooling, parent’s job), if the parent had health issues, and for parents who lacked support and/or for whom the division of the parenting roles and labour was perceived to be unequal. This therapist detailed it like this:

“*There are periods that are more challenging for families, who already have several children, two or three with challenges, and [must] work through it all. Families will have these periods which makes the parent a little less willing or available to meet with [the therapist].”* [Therapist 4].

Faced with these situations, to preserve the parent-provider relationship and the family-centered orientation of the intervention, therapists acknowledged modifying the telecoaching intervention offering more child-focused services (i.e., child in front of the computer screen completing a therapeutic activity with the therapist) and/or referring family to other services (e.g., in-person clinic visits) which better aligned with the family’s needs, expectations, and circumstances.

### Theme 3: Evolving Therapists Training Needs

In discussing their experience implementing the telecoaching intervention, therapists highlighted their training needs throughout the intervention implementation period and underscored the importance of meeting these needs in future and broader scale implementation. In their narratives, therapists described the utility of the activities and tools provided as part of their initial intervention training. Observations of a typical telecoaching session, scripts, and criteria for a successful session, regular problem-solving meetings with colleagues also using the intervention, and consultation with an expert for complex cases were deemed most helpful. While the need for some of the training tools (e.g., script, session criteria) decreased over time and with experience, telecoaching session observations, timely performance feedback, and discussions with peers and experts were deemed essential for practice change and successful implementation.

Therapists also strongly suggested carefully considering the fit of the telecoaching intervention with the family’s needs, expectations and circumstances. As such, therapists voiced a need for further skills in negotiating parent expectations, and in screening parents’ mental health and well-being. Recognizing the family-centered orientation of web-based service provision, beyond telecoaching, (e.g., for parent education, care coordination, parent peer support) within a comprehensive pediatric rehabilitation service continuum, such training was thought to be crucial in providing parents with the required information to foster shared decision-making regarding service choices.

## Discussion

This study aimed to examine the quantity and quality of a telecoaching intervention, as compared to what was intended by its creators, as well as to explore the experiences of therapists implementing it. Overall, this multimethod process evaluation indicated that the telecoaching intervention was implemented with a high degree of quality. Interestingly, our findings indicate that the key behaviours were rated similarly by the external rater and the therapists themselves. In addition, in their interviews, therapist participants detailed how the behaviours adopted in delivering the telecoaching intervention differed from approaches previously used in their practice. Some modifications to the telecoaching intervention were acknowledged by participating therapists and occurred in a response to family identified needs, expectations, and/or changing circumstances, as per family-oriented foundation of the intervention. Determining efficacy of the intervention relies on confirming the high quality of the intervention and exploring intervention modifications, including when, why, and how modifications were made (Walton et al. 2020).

A closer examination of our study findings identified some areas where the fidelity criteria may need to be further considered. Firstly, marked variations in both the session duration (24–120 minutes) and the number of sessions provided (0–21sessions) were noted. Just over half (i.e., 58%) of the families received the targeted number of telecoaching sessions in the first three months, as conceptualized by the study protocol. Many factors can influence the amount of intervention provided (e.g., the individual, the condition, their functional ability, the complexity of the goal identified, the type of intervention) (Houtrow et al., 2019; Jackman et al., 2020; Jeffries et al., 2019) and service utilization (e.g., parent and family characteristics, their knowledge about health and development, and availability and experiences with services, and their priorities) (Skeat et al., 2010).

In their interviews, participating therapists acknowledged more difficulty adhering to the telecoaching intervention components in specific circumstances, and, consequently, chose to modify the intervention. These circumstances included: (1) parents who were awaiting or had just received a diagnosis for their child (i.e., 59% of our sample); (2) parents with specific session expectations (e.g., completion of a comprehensive standardized assessment to confirm (or refute) the diagnosis; a traditional view of child-focused in-person therapy service); (3) a highly complex family contexts in which parents were overwhelmed (e.g., multiple children with exceptionalities; single-parenting; parent with chronic illness).

Interviews conducted with a purposeful sample of 16 of our parent participants as part of a related study exploring their acceptability of the intervention, underscored similar factors that impacted participating parents’ utilisation of the intervention. First, due to previous service experiences with the public health care system in their jurisdiction, some parents misunderstood the number of telecoaching sessions they could access, leading them to ceasing the interventions for fear of bothering the therapists or asking too much of them (Dostie et al., 2023). Second, parents who expected a standardized assessment, held an expectations that direct child-focused therapy delivered by the therapists was required, reported higher stress and greater mental load (Dostie et al., 2023). While some initially engaged with the intervention, disengagement occurred with the realisation that their objective in accessing these services would not be achieved, resulting in decreased use of the intervention (Dostie et al., 2023). Third, as per other studies conducted during the public health lockdowns of the COVID-19 pandemic (Dhiman et al., 2020; Seth et al., 2023), parent participants in this study highlighted difficult home and family circumstances (e.g., depressive symptoms, loss of a relative) and a lack mental and physical capacity to participate in the intervention; some even expressing regret for not using the intervention enough (Dostie et al., 2023). Parents’ expectations of an intervention, its alignment in supporting their needs, their mental health and feeling of self-efficacy have been shown to impact the rehabilitation service effectiveness (Phoenix et al., 2020; Smart et al., 2019). Future implementation of the telecoaching intervention should emphasize parents’ understanding of the service, the family context (e.g., family structure, number of children), and the need for support in providing the intervention. Moreover, establishing realistic expectations with the parents as a precursor to engaging in the intervention is critical. Finally, findings from this study and others suggest that an *a priori* decision should not be made on the dosage; instead, families’ utilisation of the intervention should be based on their needs, expectations, and priorities (Akhbari Ziegler & Hadders-Algra, 2020; Schwellnus et al., 2015; Ward et al., 2020).

Our findings also identified lower average scores relating to the therapist behaviour subscale of *WECARE Fidelity Checklist* subdomain items, in particular the identification of the parent’s goals upon which to focus the session. Collaborative goal-setting between the parents, the child (when appropriate), and the therapists is a best practice in pediatric rehabilitation and is a cornerstone of family-centered care (Pritchard-Wiart et al., 2022). Setting goals ensures that the intervention is focused on what is most meaningful to families, enhances service delivery efficiency, and improves child outcomes (Kolehmainen et al., 2012). In the current study, initial goals were set by the family with the research staff (and not the therapists), as part of determining the family’s eligibility for the study (i.e., parent able to identify at least one motor goal for their child). This research process may have hindered the collaborative nature of the goal-setting process, by initially excluding the therapists. Evidence suggests that parents value the input of therapists in the collaborative goal-setting process, shaping their goals into short-term outcomes (Pritchard-Wiart et al., 2022). From a relational perspective, therapist involvement in the decision-making process with families fosters autonomy in parents, provides emotional support to parents, and balances power relations between parents and providers (Pritchard-Wiart et al., 2019). Future telecoaching intervention implementation could consider formalizing the collaborative goal setting processes and adding associated behaviours to its assessment tools.

Despite these shortcomings, many therapist behaviours associated with family-centred care principles were rated high by the external rater. The benefits of providing family-oriented services are well-documented, including increased parent satisfaction, efficiency in the use of services, and improved child outcomes (Kuhlthau et al., 2011). High scores were assigned to items demonstrating evidence of a trusting relationship, positive rapport, and mutual respect. These items are crucial to developing strong parent-provider partnerships, a requirement for telecoaching (An & Palisano, 2014). Furthermore, items examining parents’ engagement (e.g., parents’ willingness and openness to actively participate in the session and their comfort and confidence in communicating with the therapist) were also rated high. Some studies have reported difficulties in engaging parents in web-based services (Akamoglu et al., 2018; Hines et al., 2015; Yoo et al., 2021). More recently, however, others have underscored that displaying family-centred behaviours and tailoring care to parent-identified priorities facilitate engagement in web-based service delivery models (McCarthy et al., 2021; Retamal-Walter et al., 2022). Our participating therapists reported tailoring their sessions to the needs, expectations, and changing circumstance of participating parents, even if it meant compromising the fidelity of the intervention (as described in the study protocol). This concession was not only appreciated by parents but played a crucial role in building the therapeutic alliance, which was perceived by parents and therapists as the foundation of this telecoaching intervention (Dostie et al., 2023).

Therapists’ use of a coaching style has been acknowledged as key to the success of pediatric rehabilitation web-based interventions (Graham et al., 2023). Although generally accepted to be highly collaborative, coaching approaches are not uniform, with each approach having differing assumptions, key components, and interpretations of the therapist’s role (King et al., 2024). Consensus does exist that the implementation of coaching interventions requires comprehensive and well-designed professional education, practice time, support from peers, and opportunity for reflection (Akhbari Ziegler & Hadders-Algra, 2020; King et al., 2024; Ogourtsova et al., 2019). Similarly, training has also been acknowledged as critical to the implementation of web-based interventions (Camden & Silva, 2021; Cottrell & Russell, 2020). Although this need for education and training for therapists has been acknowledged, the specific content, structure, and format for such training is rarely described in the literature (Meadan et al., 2020). Given the high-intervention fidelity scores associated with this telecoaching intervention, leveraging the training provided to the therapist participants in our study may be worth exploring in future research.

This study is not without limitations. First, although our study included all therapists who implemented the intervention, the small sample (n=4) precluded us from exploring therapist characteristics (e.g., age, therapist experience) which could impact the intervention fidelity. Secondly, the assessment instruments (e.g., the external rater and self-assessment checklists) were developed specifically for this study, as is typical (An et al., 2021), yet consequently their psychometrics were evaluated during the study (Hurtubise et al., 2024), instead of before their use. To mitigate any potential measurement limitations, a multimethod study design was chosen (Ginsburg et al., 2021). Thirdly, in this study, parent responsiveness to the intervention was evaluated by the participating therapists and by the observations of an external rater, and not by the participating parent themselves. Participants’ perspectives on their acceptability of and their experience with the telecoaching intervention were published separately (Dostie et al., 2023).

## Conclusion

Process evaluation of the implementation of an intervention is particularly important in pediatric rehabilitation interventions owing to their complexity. The novelty, changes in service delivery method, and the alternative approaches utilized, further add to the complexity of evaluating the implementation of telecoaching interventions. Unfortunately, a limited number of examples of comprehensive process evaluations exist in this field. The multimethod process evaluation presented in this manuscript aimed to fill this gap. Our study findings suggest that the telecoaching intervention was implemented with a high level of quality. In addition to documenting the quality of the telecoaching intervention implementation, this study presents key considerations on how to improve the implementation of interventions delivered using multimodal web platforms, the therapists’ training required, and the methods which can be useful in assessing it, thus filling many identified knowledge gaps in the telecoaching literature.

## Figures and Tables

**Table 1:** Participating Therapists’ Sociodemographic Characteristics

Therapist	Sex/Gender	Discipline	Education Level	Experience	Telehealth experience	Employment context	No. of families followed
1	Female	OT	Masters	7 years	0	Private	26
2	Female	PT	Doctorate	28 years	25 years	Public	27
3	Female	OT	Masters	2 years	0	Public/private	3
4	Female	PT	Bachelor	15 years	2 years	Public/private	3

*Note*. OT=occupational therapist; PT=physiotherapist

**Table 2:** Participating Families’ Sociodemographic Characteristics

Variables (n = 59)	
**Demographic - Children**	
Age (years) – mean (SD)	6.6 (1.6)
Female sex – no. (%)	18 (31)
No diagnoses – no. (%)	35 (59)
Developmental coordination disorder– no. (%)	7 (12)
Attention Deficit Hyperactivity Disorder – no. (%)	5 (9)
Developmental language disorder – no. (%)	7 (12)
Other diagnostics – no. (%)	18 (31)
Already received physiotherapy or occupational therapy services – no. (%)	
Yes, in the past, but not currently	20 (34)
No, but waiting for	16 (27)
No	23 (39)
Initial number of goals selected – median (IQR)	6 [5 – 8]
**Demographic - Parent**	
Family type – no. (%)	
Nuclear	50 (85)
Single parent	7 (12)
Extended	1 (2)
Other	1 (2)
Number of children – median (IQR)	2[2–3]
Age – no. (%)	
35 years and under	25 (42.4)
36 – 45 years	30 (50.8)
Over 45 years	4 (6.8)
Education – no. (%)	
High school	3 (5.1)
Non-university certificate or diploma (college/apprenticeship)	12 (20.3)
University certificate or bachelor’s degree	44 (74.6)
Income – no. (%)	
Less than $60,000	13/57 (22.8)
Between $60,000 and $100,000	21/57 (36.8)
Over $100,000	23/57 (40.4)
Community – no. (%)	
Urban	27 (45.8)
Suburban	22 (37.3)
Rural	10 (16.9)

**Table 3:** Quantity and Quality Dimensions, Definition, Measures, Procedures, and Analysis Summary

Dimensions	Definitions & recommended assessment	Study measurement method	Data analysis
**Intervention Quantity**			
Dose	The amount of the specific intervention delivered as measured through a count of time, frequency, or rate, operationalized as the number of treatments or the duration spent in the intervention as examined through logs or self-assessments and often compared to the intended dose set by intervention developers (An et al., 2020; Kutash et al., 2012).	Duration (in minutes) of telehealth sessions	Median number of minutes (and range) per session
		Frequency (number of sessions provided)	Median number of sessions (and range) per family
		The proportion of participants attending a telehealth session every two weeks for the first three months of the intervention (i.e., six sessions in three months), as suggested by the protocol.	Percentage of families who received six videoconferencing sessions
**Intervention Quality (Fidelity)**			
Adherence	The accurate delivery of the key intervention components as they were designed, as assessed by in-person or video-recorded observation using an instrument resembling a checklist (An et al., 2020; Kutash et al., 2012).	The score of subscale 1 (Therapists’ Behaviours) of the *WECARE Fidelity Checklist* was rated by an external rater based on their monthly assessment of video-recorded observations of a telehealth session.	Median score frequency distribution of the 12 Therapist Behaviours of subscale 1(i.e., score out of 60)
		Therapist completion of a self-assessment survey of three target behaviours (session focus, parent engagement, relationship building), rated on a 5-point Likert scale following all recorded telehealth sessions conducted during a selected week each month.	Median score and frequency distribution of the three targeted behaviours (i.e., score out of 15)
Participant responsiveness	The extent to which participants engaged in the intervention’s components and activities and/or are satisfied with the intervention; can be assessed through observation of participants’ behaviors if they respond as expected or through their self-report of satisfaction with the intervention (An et al., 2020; Breitenstein et al., 2010; Kutash et al., 2012).	The score of subscales 2 (Parent Behaviours) and 3 (Parent-Therapist Interaction) of the *WECARE Fidelity Checklist* as rated by external raters based on their monthly assessment of videorecorded observations of a telehealth session	Median score and frequency distribution of four Parent Behaviours of subscale 2 (i.e., score out of 20) and the two of Parent-Therapist Interaction Behaviours of subscale 3 (i.e., score out of 10)
		Parents’ satisfaction ratings with the intervention as assessed at the end of the intervention using nine statements rated on a 5-point Likert scale (1=do not at all agree; 5=completely agree). The statements captured the usefulness of the WECARE intervention in meeting parent/family needs	Median score and frequency distribution of ratings (i.e., score out of 45)
The overall quality of the intervention	How therapists use the overall strategies or processes developed by the study designer and often rely on the judgement (based on observation) of an intervention expert (An et al., 2020; Kutash et al., 2012; O’Donnell, 2008).	Number of sessions where 75% of the *WECARE Fidelity Checklist* behaviours were scored as a “4 or 5” by the external rater	Frequency of the number of sessions.

**Table 4:** Fidelity Checklist Scoring System

Score	External Rater description qualifiers
1	The behaviour was not observed when it should have been.
2	The behaviour is observed at a limited frequency and/or is only partially observed.
3	The behaviour is observed at times, but several opportunities to use this behaviour are missed.
4	The behaviour is observed regularly, but not all opportunities are seized.
5	The behaviour is always observed when appropriate (i.e., is applied as intended in the development of the intervention and observed at the appropriate time).

**Table 5:** Summary of the Quantity (Dosage) and Quality (Fidelity) Dimensions Results

Dimension	Measures	Median score (Range)
**Intervention Quantity**		
Dosage	Session duration	39 minutes (24, 120 minutes)
	Frequency	9 sessions (0, 21 sessions)
	Number of families who received the anticipated number of sessions (n=6) during the first 3 months	34/59
**Intervention Quality (Fidelity)**		
Adherence	External ratings of therapist behaviours subscale	50/60 (46, 57)
	Therapists’ participant self-assessment	13/15 (10, 13)
Participant responsiveness	External rating of parent behaviour subscale	18/20 (12, 20)
	External rating of parent-therapist interaction subscale	10/10 (6, 10)
	Parent participants’ satisfaction with the intervention	45/45 (7, 45)
The overall quality of intervention	Number of sessions where 75% of the checklist behaviours were scored as a “4 or 5” by the external rater	23/33

**Table 6:** Themes Emerging from Therapists’ Experience Implementing WECARE

Theme		Focus	Description	Implications
1.	Increased collaboration with parents	How intervention differed from previous approached	Therapists reported greater partnership and shared decision-making with families compared to traditional therapist-directed sessions.	A shift toward a family-centered, coaching model emphasizing joint goal setting and parent empowerment is required.
2.	Remaining family-oriented	Interventions modifications and their rationale	Adaptations were made to meet family needs, such as flexible scheduling, tailored resources, and accommodating home contexts.	Contextual responsiveness and individualized care to maintain family engagement is vital.
3.	Evolving therapists’ needs	Recommendations for future implementation	Therapists identified new skill requirements (e.g., coaching techniques, technology use, family engagement strategies) to sustain and scale telecoaching.	Training frameworks and professional development are required to support fidelity in scaling this intervention more broadly.

## Appendix A Interview Guide Script – Participating Therapist

### Introduction

First, I would like to thank you for participating in the project and for agreeing to take the time to share with me your experience implementing the intervention.

The purpose of the interview is to get your opinion on the intervention proposed in the WECARE project. The questions will focus on your perception of the usefulness of the WECARE intervention, your experiences implementing it, and your recommendations for potential improvements of a tele-coaching intervention such as WECARE.

The interview lasts approximately one hour and will be recorded for research purposes. The results will remain confidential. The recording of your interview will be destroyed at the end of the project. The transcript of the interview will be kept for a period of 10 years like all other data in the research project.

Do you have any questions before starting the interview?

1. **Can you tell me about your experience with the WECARE intervention?**
  - What did you like most and least about this intervention?
  - What did you find most challenging about implementing this intervention?
  - Did you have any negative experiences? If so, can you describe them?
  - Did you notice any factors (positive and/or negative) that had an impact on the implementation of the WECARE intervention?
  - Did you have to change any anything?
2. **Before starting the WECARE intervention, how would you describe your level of confidence in performing a telecoaching intervention?**
  - What do you think of the training you were given to prepare for the procedure?
  - How would you describe the evolution of your coaching skills through the intervention?
  - How were your training needs met or not during the WECARE intervention?
  - What activities have (or would have) been most useful to support this evolution or your needs during the WECARE intervention?
  - How would you describe the changes in your practices?
3. **What do you think of the telecoaching method?**
  - What do you think of its effectiveness?
  - What do you think of its usefulness?
4. **What were the impacts of the WECARE intervention that you have observed?**
  - In children?
  - In parents or families?
  - In access to services?
  - In your practice?
  - In other areas?
5. **What do you think of the self-evaluation quality questionnaire?**
  - If applicable, how did this questionnaire impact your behaviours, attitudes, abilities as a worker?
6. **Has the WECARE intervention changed the way you think about how pediatric rehabilitation and child development services are delivered? If so, can you share an example with me**.
7. **Has the WECARE intervention changed the way you provide services to families? If so, can you share an example with me**.
8. **Do you have any recommendations for the WECARE intervention if it was to be implemented into the broader healthcare system?**
  What support would therapists need to implement and sustain this type of intervention?
9. **Do you have any other comments or questions related to the WECARE intervention and your experience with implementing it?**

Thank you for your participation!

End of the recording.
